# Relationship between the Corticospinal and Corticocerebellar Tracts and Their Role in Upper Extremity Motor Recovery in Stroke Patients

**DOI:** 10.3390/jpm11111162

**Published:** 2021-11-08

**Authors:** Jungsoo Lee, Won Hyuk Chang, Yun-Hee Kim

**Affiliations:** 1 Department of Physical and Rehabilitation Medicine, Center for Prevention and Rehabilitation, Heart Vascular Stroke Institute, Samsung Medical Center, Sungkyunkwan University School of Medicine, Seoul 06351, Korea; jungsoo0319@gmail.com (J.L.); wh.chang@samsung.com (W.H.C.); 2 Department of Health Sciences and Technology, Department of Medical Device Management & Research, Department of Digital Health, SAIHST, Sungkyunkwan University, Seoul 06351, Korea

**Keywords:** stroke, motor recovery, corticospinal tract, corticocerebellar tract

## Abstract

The corticospinal tract (CST) and corticocerebellar tract (CCT) are both involved in the upper extremity (UE) function after stroke. Understanding the relationship between the tracts and their functions can contribute to developing patient-specific rehabilitative strategies. Seventy ischemic stroke patients who underwent diffusion tensor imaging (DTI) two weeks after the stroke onset and motor function assessments two weeks and three months after the stroke onset were included in this study. To obtain the CST and CCT integrity, the functional anisotropy (FA) values of both tracts were extracted from the DTI data. Linear regression was used to identify the relationship and predictive accuracy. The CST FA data had predictive values, but CCT FA did not. There were interaction effects between the CST and CCT FA values (*p* = 0.011). The CCT was significantly associated with high CST FA but not low CST FA. When the CST or CCT FA were applied to patients depending on the CST status, the stratified model showed higher predictive accuracy (*R*^2^ = 0.380) than that of the CST-only model (*R*^2^ = 0.320). In this study, the conditional role of CCT depending on CST status was identified in terms of UE recovery in stroke patients. This result could provide useful information about individualized rehabilitative strategies in stroke patients.

## 1. Introduction

Motor recovery is crucial in stroke rehabilitation because motor function deficits strongly affect daily life activities and mobility. Motor recovery usually occurs in the first three months following a stroke but inter-individual variability exists during the recovery period [1,2]. Understanding motor recovery considering patient characteristics can provide clinicians with important information related to individualized treatment [3].

The corticospinal tract (CST), which is the pyramidal tract, originates primarily from the motor-related cortical regions, including the primary motor cortex, secondary motor area, premotor cortex, and somatosensory cortex, through the posterior limb of the internal capsule (PLIC) and cerebral peduncle (CP) to the spinal cord [4]. The CST is a major neural pathway that primarily carries movement-related information and mediates voluntary movements. The corticocerebellar tract (CCT), which is a sensorimotor pathway, reciprocally connects the motor-related regions and cerebellum. The CCT tract comprises descending and ascending tracts; the ascending tract via the superior cerebellar peduncle (SCP) and midbrain to contralateral motor-related regions comprise the majority of the tracts [5]. The CCT is associated with motor learning, especially fine motor skills [6,7,8].

Neuroimaging studies have been performed to investigate the predictive biomarkers of motor recovery after stroke, and both tracts have been designated as candidates of imaging biomarkers [9,10,11]. Particularly, the extent of CST damage is the best representative biomarker of recovery of the upper extremity (UE) after a stroke [9]. The CST lesion load, which is calculated by overlaying the lesion on imaging with CST template, and the CST integrity, which is the fractional anisotropy (FA) value of the CST region using diffusion tensor imaging (DTI), are representative techniques to investigate the extent of CST damage in stroke patients [12,13,14]. The smaller the CST lesion load and the larger the CST FA value at acute and subacute phase, the better the motor recovery. In addition, previous neuroimaging studies reported that the CCT plays an important role in motor function using DTI and resting-state functional magnetic resonance imaging (fMRI) data [15,16,17]. The CCT FA value and cerebellar functional connectivity were positively associated with motor outcome. 

Among neuroimaging techniques, the FA value, obtained from DTI data, means the degree of anisotropy of water molecules in the specific tract. This scalar value between zero and one is related to fiber density, axonal diameter, and myelination [18]. This FA value has been used to examine white matter integrity in many clinical neuroimaging studies [10,19,20]. After stroke onset, the injured tract region’s FA value generally decreases compared to intact tract regions due to Wallerian degeneration in the injured tract region [21]. Relatively high FA values mean low damage of the specific tract. Therefore, this technique can deliver more information about white matter damage than investigating the lesion location using anatomical imaging data.

The CST and CCT are crucial sensorimotor tracts for motor function in stroke patients, but their relationship has not been fully investigated. Clarifying the relationship between important markers not only provides information about recovery mechanisms after a stroke but also leads to more accurate recovery predictions. Moreover, new results from these approaches can be used to develop individualized treatment strategies. In the present study, we investigated the relationship between the CST and CCT in terms of UE motor recovery using FA analysis obtained from the DTI data of subacute stroke patients. This study examined the specific or conditional roles of the two tracts during the subacute recovery period in stroke patients.

## 2. Materials and Methods

### 2.1. Study Participants

One hundred and four ischemic stroke patients with motor impairment in the subacute stage were screened retrospectively from the stroke database at the Department of Physical and Rehabilitation Medicine, Samsung Medical Center. Most patients received early rehabilitation for progressive mobilization within one week after onset and started comprehensive inpatient rehabilitation therapy around two weeks after stroke onset. These patients received comprehensive inpatient rehabilitation therapy for approximately three weeks. The inclusion criteria were as follows: (1) age 19 years or older at stroke onset, (2) experiencing the first-ever unilateral stroke, (3) demonstrating severe or moderate motor impairment (Fugl–Meyer Assessment (FMA) of UE score < 35 at two weeks) [22], and (4) undergoing diffusion tensor imaging (DTI) at two weeks after onset. The exclusion criteria were as follows: (1) clinically significant neuropsychiatric comorbidity, (2) history of metallic implants in the brain, and (3) bilateral lesions. Seventy stroke patients (42 male and 28 female patients with an average age of 58.1 ± 12.9 years) were included in this analysis. Table 1 summarizes the demographic and clinical characteristics of the participants. The patients underwent initial clinical assessments, including the FMA, National Institutes of Health Stroke Scale (NIHSS), and Mini Mental State Examination (MMSE), before starting comprehensive inpatient rehabilitation therapy at two weeks after stroke onset. The FMA examination was repeated three months after stroke onset to examine the motor recovery in the subacute phase.

Ethical approval was obtained from the Institutional Review Board (IRB) of Samsung Medical Center in Seoul, Republic of Korea. We received an exemption for informed consent from the IRB because we used retrospective data and did not exceed the minimal risk.

### 2.2. MRI Data Acquisition

The DTI and T1-weighted structural data were acquired using a 3-Tesla (3T) Philips ACHIEVA^®^ MR scanner (Philips Medical Systems, Best, The Netherlands). For DTI data, 60 axial slices were obtained covering the whole brain with gradients (b = 1000 s/mm^2^) applied along 45 non-colinear gradient directions with the following settings: slice thickness = 2.25 mm, no gap, matrix size = 112 × 112, repetition time = 8770 ms, echo time = 60 ms, and field of view = 220 × 220 mm. For T1-weighted structural imaging, 124 axial slices were obtained covering the whole brain with the following settings: slice thickness = 1.6 mm, no gap, matrix size = 512 × 512, repetition time = 13.9 ms, echo time = 6.89 ms, flip angle = 8°, and field of view = 240 × 240 mm.

### 2.3. DTI Data Processing for Extraction of CST and CCT FA Values

**Figure 1:**
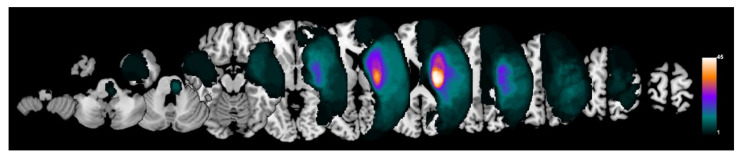
Lesion maps. The stroke lesions were flipped for patients with lesions on the right side. All lesions were overlaid on the left side.

Individual lesions were manually drawn by a medical doctor on the diffusion-weighted images using FSL view 4.0.1 software (part of FSL software version 5.0.9, FMRIB, Oxford, UK, http://www.fmrib.ox.ac.uk/fsl, accessed on 15 June 2019). Each lesion volume image was warped to the Montreal Neurological Institute (MNI) standard space to investigate the lesion distribution of participants. All lesions were overlaid on the left side by flipping the images horizontally for patients with lesions on the right side to visualize the lesion distribution. The results were visualized using MRIcroGL (McCausland Center for Brain Imaging, University of South Carolina, http://www.cabiatl.com/mricrogl, accessed on 7 March 2020) (Figure 1). The lesion volume was calculated by counting the number of lesioned voxels on the normalized individual lesion volume image in MNI standard space and multiplying the number of lesioned voxels by the volume of each voxel. 

**Figure 2:**
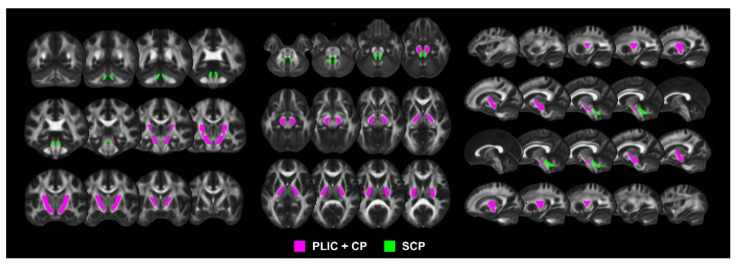
PLIC, CP, and SCP regions extracted from the JHU ICBM-DTI-81 Atlas. PLIC, posterior limb of the internal capsule; CP, cerebral peduncle; SCP, superior cerebellar peduncle.

DTI data processing was performed using the FMRIB Diffusion Toolbox in the FSL software package, version 5.0.9 (FMRIB Software Library, FMRIB, Oxford, UK, http://www.fmrib.ox.ac.uk/fsl, accessed on 15 June 2019). Corrections for eddy currents produced by the diffusion-sensitizing gradients and head motion during scan time and skull stripping were processed. The *DTIfit* algorithm was used to calculate the FA maps from the preprocessed DTI data. Individual FA maps were registered non-linearly to the MNI standard space (FMRIB58_FA standard space image) using the tract-based spatial statistics technique to overlay all FA maps in the standard space. During registration, the lesioned voxels were masked. To obtain the CST and CCT FA values from the registered FA maps in the standard space, the JHU ICBM-DTI-81 Atlas [23], which is parcellated white matter tracts of the whole brain into 48 tract regions, was used. The PLIC, CP, and SCP regions were extracted from the Atlas in the standard space. The FA value in the combined PLIC and CP regions and the FA value in the SCP region were used as the CST and CCT integrity values, respectively (Figure 2). The mean FA values of the regions were calculated by averaging all FA values in the CST and CCT regions, and the proportional CST FA value was calculated as the ratio of the ipsilesional value to the contralesional value. Since the affected and unaffected SCP are located inversely by tract decussation compared to the PLIC and CP, the CCT FA value was calculated as the ratio of the contralesional value to the ipsilesional value to ensure the polarity of the relationship between the degree of tract damage and motor recovery.

### 2.4. Statistical Analysis

All variables were assessed for a normal distribution using the Shapiro–Wilk normality test. Linear regression was used to identify the predictive values of DTI tracts for the improvement of FMA-UE. Multiple regression analysis was used to examine the interaction effects of the CST and CCT FA values on the improvement in FMA-UE. Additionally, the predictive values of the CCT FA were investigated in subgroups divided according to the cutoff of 0.9 for proportional CST FA, which separates between intact and injured CST [24,25,26,27]. The predictive accuracy was represented as *R*^2^ in this study. The statistics were derived using the *fitlm* and *anova* functions in the statistics toolbox of MATLAB R2014b. In this study, *p* < 0.05 was considered statistically significant.

## 3. Results

In univariate analysis, the CST FA showed a significantly positive correlation with FMA-UE recovery (*R*^2^ = 0.320, *p* < 0.001), while the CCT FA did not (*R*^2^ = 0.004, *p* = 0.620). The higher the CST FA, the better the FMA-UE recovery was. In multivariate analysis, both CST and CCT FA values were significant (CST, *p* = 0.032; CCT, *p* = 0.018) with a significant interaction effect (CST*CCT, *p* = 0.011) (Table 2). The relationship between one tract FA value and FMA-UE recovery depended on another tract FA value.

**Figure 3:**
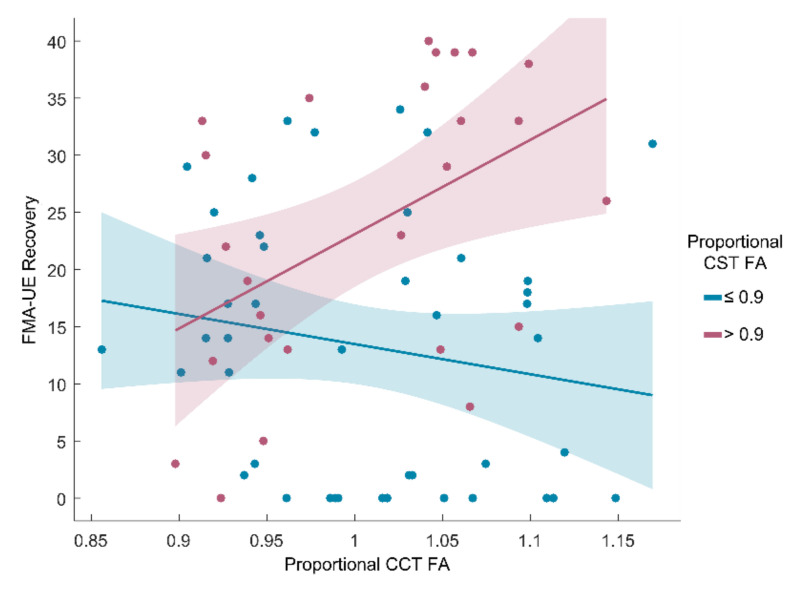
Relationship between the CCT FA and FMA-UE recovery in subgroups divided by CST integrity (proportional CST FA cutoff = 0.9). The CCT FA was significant in patients with high CST FA (*n* = 26, *R*^2^ = 0.223, *p* = 0.015) but not in patients with low CST FA (*n* = 44, *R*^2^ = 0.031, *p* = 0.252). FMA-UE, Fugl–Meyer Assessment-Upper extremity; CST, corticospinal tract; CCT corticocerebellar tract.

Based on the interaction effect, a subgroup analysis was performed according to the CST integrity of the patients. The CCT FA had predictive values in patients with high CST FA (proportional CST FA > 0.9) but not in patients with low CST FA (proportional CST FA ≤ 0.9) (Figure 3). The higher the CCT FA, the better the FMA-UE recovery was in patients with high CST FA. Conversely, the CST FA was not significant in patients with high CST FA, but it showed high predictive accuracy in patients with low CST FA (Table 3). The higher the CST FA, the much better the FMA-UE recovery was in patients with low CST FA.

Finally, a prediction model using a stratification strategy was proposed. A stratified model was created by the cutoff of the CST FA of stroke patients; the CCT FA was used as the predictive factor when the CST FA was higher than 0.9, whereas the CST FA was used as the predictive factor when the CST FA was less than or equal to 0.9. This stratified model (*R*^2^ = 0.380, *p* < 0.001) showed a higher predictive accuracy than that of the CST-only model (*R*^2^ = 0.320, *p* < 0.001).

## 4. Discussion

In this study, using DTI data of subacute stroke patients, we investigated the roles of the CST and CCT, as well as their relationship with regard to UE motor recovery. The degree of CST damage was significantly related to UE recovery in all patients, but that of the CCT was not. However, the CCT was significant when CST damage was relatively less. This means that while the CST is the primary biomarker, the CCT acts as a secondary biomarker for UE motor recovery depending on the status of the CST.

### 4.1. The Corticospinal Tract Injury Associated with Motor Recovery

The predictability of CST injury after stroke has been validated in many studies [12,13,14]. The PLIC integrity obtained from DTI data is the most significant predictive biomarker of UE motor outcome, which has been used in algorithms that predict motor recovery [28,29]. The CST lesion load, which measures the overlapping volume of the CST and lesion using anatomical imaging data, correlates well with motor impairment and recovery [12]. In addition, both the presence and absence of motor-evoked potentials (MEPs), which measure the integrity of the CST using transcranial cortical stimulation with a magnetic coil, are effective predictive markers for a satisfactory or poor recovery, respectively [3]. The degree of CST damage is determined to be crucial in predicting UE recovery. Furthermore, less damage to the CST is related to improvements in treatment gains [30,31]. For these reasons, the CST is well known as a representative biomarker based on expert consensus [9]. In our study, the CST also played a significant role in predicting UE motor recovery.

### 4.2. The Corticocerebellar Tract Injury Associated with Motor Recovery

The CCT is a sensorimotor tract that contributes to motor learning, control, and the acquisition of motor memory [6,7]. Using DTI data, a significant association between CCT and residual motor output has been reported in chronic stroke patients. [16]. Several fMRI studies have reported that cerebellar functional activity and functional connectivity are positively related to motor outcome [32,33,34]. An MEP study found that motor cortical excitability was reduced in cerebellar stroke patients [35]. However, the number of CCT studies related to motor recovery is sparse compared to the number of CST studies, despite the CCT being a vital region involved in motor functions. Some studies have indicated that the cerebellum is not related to motor outcome and recovery, based on whole-brain analysis using DTI and resting-state fMRI data [36,37]. Thus, the CCT has an inconsistent role in predicting UE recovery in stroke patients.

### 4.3. The Relationship between the Corticospinal and Corticocerebellar Tracts for Motor Recovery

In our study, the CCT data were significant in patients with high CST integrity but not in patients with low CST integrity. In other words, the CCT played an important role in UE recovery when the CST was relatively preserved. Considering that the CCT is related to fine motor control [6,7,8], it might contribute to the recovery of fine motor skills with preserved CST. Although different brain regions are involved in the motor recovery process after a stroke according to the initial severity of the stroke, lesion location, CST integrity, etc. [15,38,39], the role of neural connections can vary depending on the degree of CST damage. Investigating the conditional role of specific neural connections can provide useful information for understanding the motor recovery mechanism within the human brain. According to previous studies, the structure and function of the CCT are more complicated than those of the CST. The corticospinal pathway is connected primarily to cortical motor-related regions, while the corticocerebellar pathway has a larger proportion of its structure in the prefrontal cortex than in the cortical motor-related regions [40]. The cerebellum is involved in both cognitive and motor functions [41,42]. Clinical, neuroimaging, and neurophysiological evidence suggest that the cerebellum participates in executive, spatial cognitive, and language functions [41]. There are many theoretical models of the functional role of the cerebellum. Therefore, it is difficult to explain systematically how common cerebellar mechanisms may contribute to both motor control and cognitive function [40]. In our study, the CST data were not significant for patients with high CST integrity. In other words, in patients who experienced less CST damage, the CST integrity might not have enough discrimination power for further UE recovery prediction. However, this relationship does not indicate that the CST is not important in patients with less CST damage. In this study, we proposed a prediction model using a stratification strategy. When the patients’ UE motor recovery model was created using both CST and CCT FA stratified by their CST status, the proposed model showed higher predictive accuracy than the CST-only model.

### 4.4. Conclusions

In conclusion, we investigated the relationship between the CST and CCT in terms of UE motor recovery using DTI data from subacute stroke patients. Although our study could not completely identify the role of the CCT in UE recovery, it uncovered important information on the conditional role of CCT depending on CST status during the subacute stroke period. In addition, we created a better UE recovery prediction model by using both CST and CCT FA. These results will be helpful in developing patient-specific rehabilitative strategies, such as individualized target areas of noninvasive brain stimulation according to the CST status of subacute stroke patients. 

## Figures and Tables

**Table 1:** Demographic and clinical characteristics of participants.

Characteristics	*n* = 70
Age (years)	
Mean ± SD	59.1 ± 12.9
Sex (*n*)	
Male	42
Female	28
Lesion side (*n*)	
Right	34
Left	36
Lesion location (*n*)	
Supratentorial	57
Infratentorial	13
Lesion volume (cm^3^)	
Mean ± SD	49.9 ± 78.1
Initial impairment, mean ± SD	
FMA-UE	13.8 ± 9.4
NIHSS	8.9 ± 4.4
MMSE	23.0 ± 9.1

SD, standard deviation; FMA-UE, Fugl–Meyer Assessment-Upper extremity; NIHSS, National Institutes of Health Stroke Scale; MMSE, Mini Mental State Examination.

**Table 2:** Multivariate model of the upper extremity motor recovery in all participants.

Model	Estimate	*t*	*p*	*R* ^2^	*Adjusted R* ^2^
CST*CCT				0.358	0.329
Intercept	281.42	2.12	0.038		
CST	−340.6	−2.19	0.032		
CCT	−313.5	−2.44	0.018		
CST*CCT	397.9	2.63	0.011		

CST, corticospinal tract; CCT, corticocerebellar tract.

**Table 3:** Predictive values in subgroups divided by CST integrity.

Tracts	Proportional CST FA > 0.9 (*n* = 26)			Proportional CST FA ≤ 0.9 (*n* = 44)		
	*t*	*p*	*R* ^2^	*t*	*p*	*R* ^2^
CST	−0.89	0.382	0.032	4.75	<0.001	0.349 *
CCT	2.63	0.015	0.223 *	−1.16	0.252	0.031

CST, corticospinal tract; FA, fractional anisotropy; CCT corticocerebellar tract (* *p* < 0.05).

## Data Availability

The data that support the findings of this study are available from the corresponding author upon reasonable request.
